# A new concordant partial AUC and partial c statistic for imbalanced data in the evaluation of machine learning algorithms

**DOI:** 10.1186/s12911-019-1014-6

**Published:** 2020-01-06

**Authors:** André M. Carrington, Paul W. Fieguth, Hammad Qazi, Andreas Holzinger, Helen H. Chen, Franz Mayr, Douglas G. Manuel

**Affiliations:** 1 Ottawa Hospital Research Institute, Ottawa, K1H 8L6 Canada; 2 Faculty of Engineering, University of Waterloo, Waterloo, N2L 3G1 Canada; 3 Holzinger Group (HCAI), Institute for Medical Informatics/Statistics, Medical University Graz, 8036 Graz, Austria; 4 Institute of Interactive Systems and Data Science, Graz University of Technology, 8010 Graz, Austria; 5 School of Public Health and Health Systems, University of Waterloo, Waterloo, N2L 3G1 Canada; 6 Universidad ORT Uruguay, 11100 Montevideo, Uruguay; 7 Department of Family Medicine, University of Ottawa, Ottawa, Canada; 8 School of Epidemiology, Public Health and Preventive Medicine, University of Ottawa, Ottawa, Canada; 9 Institute for Clinical Evaluative Sciences, Ottawa, Canada; 10 Statistics Canada, Ottawa, Canada; 11 C.T. Lamont Primary Health Care Research Centre and Bruỳere Research Institute, Ottawa, Canada; 12 Division of Clinical Public Health, Dalla Lana School of Public Health, Toronto, Canada

**Keywords:** Area under the ROC curve, Receiver operating characteristic, C statistic, Concordance, Partial area index, Imbalanced data, Prevalence, Classification, Diagnostic testing, Explainable artificial intelligence

## Abstract

**Background:**

In classification and diagnostic testing, the receiver-operator characteristic (ROC) plot and the area under the ROC curve (AUC) describe how an adjustable threshold causes changes in two types of error: false positives and false negatives. Only part of the ROC curve and AUC are informative however when they are used with imbalanced data. Hence, alternatives to the AUC have been proposed, such as the partial AUC and the area under the precision-recall curve. However, these alternatives cannot be as fully interpreted as the AUC, in part because they ignore some information about actual negatives.

**Methods:**

We derive and propose a new concordant partial AUC and a new partial *c* statistic for ROC data—as foundational measures and methods to help understand and explain parts of the ROC plot and AUC. Our partial measures are continuous and discrete versions of the same measure, are derived from the AUC and c statistic respectively, are validated as equal to each other, and validated as equal in summation to whole measures where expected. Our partial measures are tested for validity on a classic ROC example from Fawcett, a variation thereof, and two real-life benchmark data sets in breast cancer: the Wisconsin and Ljubljana data sets. Interpretation of an example is then provided.

**Results:**

Results show the expected equalities between our new partial measures and the existing whole measures. The example interpretation illustrates the need for our newly derived partial measures.

**Conclusions:**

The concordant partial area under the ROC curve was proposed and unlike previous partial measure alternatives, it maintains the characteristics of the AUC. The first partial c statistic for ROC plots was also proposed as an unbiased interpretation for part of an ROC curve. The expected equalities among and between our newly derived partial measures and their existing full measure counterparts are confirmed. These measures may be used with any data set but this paper focuses on imbalanced data with low prevalence.

**Future work:**

Future work with our proposed measures may: demonstrate their value for imbalanced data with high prevalence, compare them to other measures not based on areas; and combine them with other ROC measures and techniques.

## Background

The ability of a classifier or diagnostic test to discriminate between actual positives and negatives, is often assessed by its curve in a receiver-operator characteristic (ROC) plot and the area under the ROC curve (*AUC*). However, when data are imbalanced with few positives relative to negatives (i.e. a low prevalence or incidence of a disease in the total population), we need high specificity to avoid a large number of false positives and ideally high sensitivity as well. For example, the prevalence of breast cancer in Western Europe is 90 per 100,000 women per year (*<* 0*.*01%) [1]; hence, a screening test with 100% sensitivity and 99.9% specificity will have 90 false positives for every 10 true positives. The *AUC* does not focus on the need for high specificity in the leftmost part of an ROC curve.

Two strategies are used to address limitations of the ROC and *AUC* in a low prevalence setting—the partial area under the ROC curve (*pAUC*), or using a different plot, the precision-recall curve and its associated area under the PRC (AUPRC), also called average precision (AP). Neither strategy fully represents the information in the part of the curve that is of interest.

This study outlines limitations of the *pAUC* and AUPRC, reviews related work and then derives new measures to address those limitations. It derives the partial *c* statistic for ROC (*c*_*∆*_) and other measures with the end goal of deriving the concordant partial *AUC* (*pAUC*_*c*_). We then perform experiments to validate the correctness of the measures and provide interpretation for some results.

**Fig. 1:**
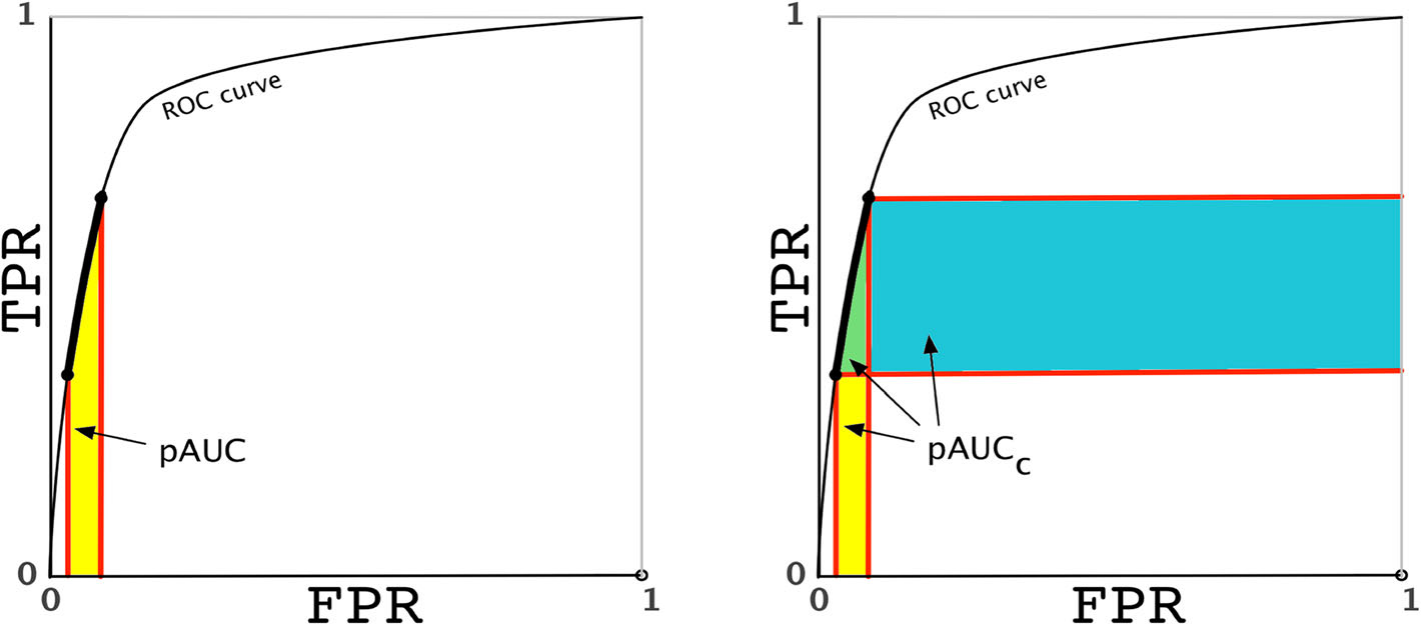
The partial *AUC* versus our proposed concordant partial *AUC.* **a** The partial *AUC* (*pAUC*) provides a vertical perspective that represents the average TPR for part of the ROC curve (thick line) multiplied by the horizontal width. **b** The concordant partial *AUC* (*pAUC*_*c*_) combines vertical and horizontal perspectives and equals the partial *c* statistic

**Fig. 2:**
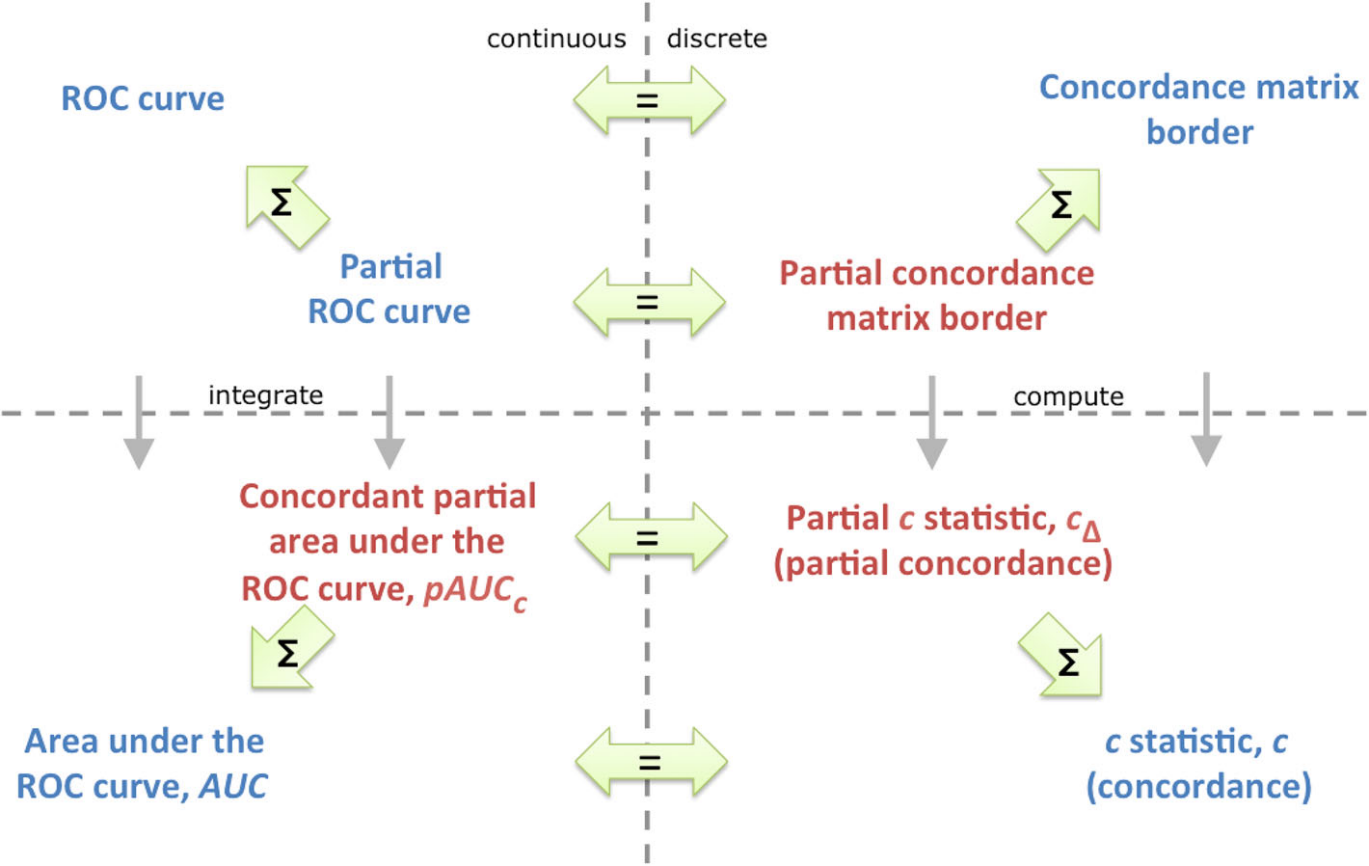
An overview of our proposed measures and concepts (red). For a set of partial ROC curves which span the whole curve, without overlap, the sum ∑ of partial measures/concepts equals the whole measure; and the continuous ROC/*AUC* concepts equal their discrete c statistic and concordance matrix counterparts

There are a rich set of relationships between our proposed partial measures and the existing whole measures and a fair bit of background, so we provide an overview of our measures (Figs. 1 and 2) and their definitions (Table 1) as context for the related work and review sections that follow. Our measures resolve issues and offer greater understanding and explanation for partial areas in ROC plots.

**Table 1:** An overview of definitions for proposed measures and concepts in sections that follow with the same name

1. The horizontal partial area under the curve (a section that follows) This partial area denoted *pAUC*_*x*_, was suggested by Walter [2] and is defined for part or an ROC curve *r*(·) defined by *TPR* = [*y*1*, y*2] with inverse function *r*^−1^(·): ${pAUC}_x:={\int}_{y_1}^{y_2}1-{r}^{-1}(y) dy$	
2. The concordant partial area under the curve (a section that follows) This partial area denoted *pAUC*_*c*_ (Fig 1b) is defined for part of an ROC curve *r*(·) defined by *FPR* = [*×* 1*, ×* 2] and *TPR* = [*y*1*, y*2], with inverse function *r*^−1^(·): $$ {\displaystyle \begin{array}{c} pAU{C}_c\triangleq \frac{1}{2} pAU C+\frac{1}{2} pAU{C}_x\\ {}=\frac{1}{2}{\int}_{x_1}^{x_2}r(x) dx+\frac{1}{2}{\int}_{y_1}^{y_2}1-{r}^{-1}(y) dy\end{array}} $$	
3. The concordance matrix for ROC data (a section that follows) A matrix that depicts the exact relationship between the unique scores of positives and negatives in data and their corresponding points along a matrix border that exactly matches the (empirical) ROC curve. It geometrically and procedurally equates area measures *AUC* and *pAUC*_*c*_ to the statistics *c* and *c*_*∆*_.	
4. The partial *c* statistic for ROC data (a section that follows) This statistic denoted *c*_*∆*_ is defined for ROC data with *P* actual positives {*p*_1…*P*_} and *N* actual negatives {*n*_1…*N*_} and a partial curve specified by a subset of *J* positives and *K* negatives, i.e., $\left\{{p}_{1\dots J}^{\prime}\right\}$ and $\left\{{n}_{1\dots K}^{\prime}\right\}$; with Heaviside function *H*(·) and classification scores *g*(·). We present simple *c*_*∆*_ (the non-interpolated version) here: $$ {\displaystyle \begin{array}{c}\mathrm{simple}\ {\mathrm{c}}_{\varDelta}\triangleq \frac{1}{2 JN}\sum \limits_{j=1}^J\sum \limits_{k=1}^NH\left(g\left({p}_j^{\prime}\right)-g\left({n}_k\right)\right)\\ {}+\frac{1}{2 PK}{\sum}_{j=1}^P{\sum}_{k=1}^KH\left(g\left({p}_j\right)-g\left({n}_k^{\prime}\right)\right)\end{array}} $$	

A Receiver Operator Characteristic (ROC) plot [3–5] depicts how a classifier or diagnostic test performs or errs at different thresholds. It may depict a curve which is fit to data (Fig 1), or a plot which exactly represents the data called an empirical ROC plot (Fig 4b) or a convex polygon, called an ROC convex hull [6] which represents the performance possible by interpolating between one classifier at two thresholds (hence not the original classifier itself) or between two classifiers. We refer to all three as “ROC curves”.

The area under the ROC curve (*AUC*) represents the ability of the classifier (or test) to produce a higher score for an actual positive than an actual negative— i.e., the (underlying) ability to discriminate positives from negatives according to the score (properly called a classification score). This interpretation of the *AUC* is known as the *c* statistic or concordance [7–10], and the two are equal *AUC* = *c* for binary outcomes—excluding survival or “censored” data, with outcomes that include time-to-event.

Two other interpretations [11] of the *AUC* are that it represents the average true positive rate (TPR) a.k.a. average sensitivity, over all thresholds or all specificity values; and it represents the average true negative rate (TNR) a.k.a. average specificity, over all thresholds or all sensitivity values.

### Review of the partial area under the ROC curve (*pAUC*)

For an ROC curve *y* = *r*(*x*), the partial area under the ROC curve (*pAUC*) [12, 13].

1 $$ pAUC\triangleq {\int}_{x_1}^{x_2}r(x) dx $$

allows us to focus on the area of interest on the left side of the ROC plot (Fig 1a) and avoid the region of high false positives to the right, which may not be relevant [14, 15], or which may not be clinically acceptable [2]. That is, the *pAUC* addresses some criticisms of the *AUC*.

McClish [12] uses the partial *AUC* on published data [16] for paired ROC curves in computed tomography (CT) examinations with and without clinical history by one individual. McClish [12] showed that when two curves are compared in the false positive range of 0 to 10% rather than a specific threshold of 10%, the results were significantly different in the latter case but not the former. However, the author [12] does not provide a clinical interpretation of the results.

While the *pAUC* may improve upon the *AUC*, it does not fully represent the partial curve that is of interest. Walter [2] expresses concern that the *pAUC* is not symmetric in its consideration of positives and negatives in contrast to the *AUC*. It ignores actual negatives (whether false positives or true negatives), except as bounds on the region of interest. Furthermore, *pAUC* lacks a defined relationship to the *c* statistic (concordance), which gives concrete meaning to *AUC* values, and which is also symmetric in its perspective.

**Fig. 3:**
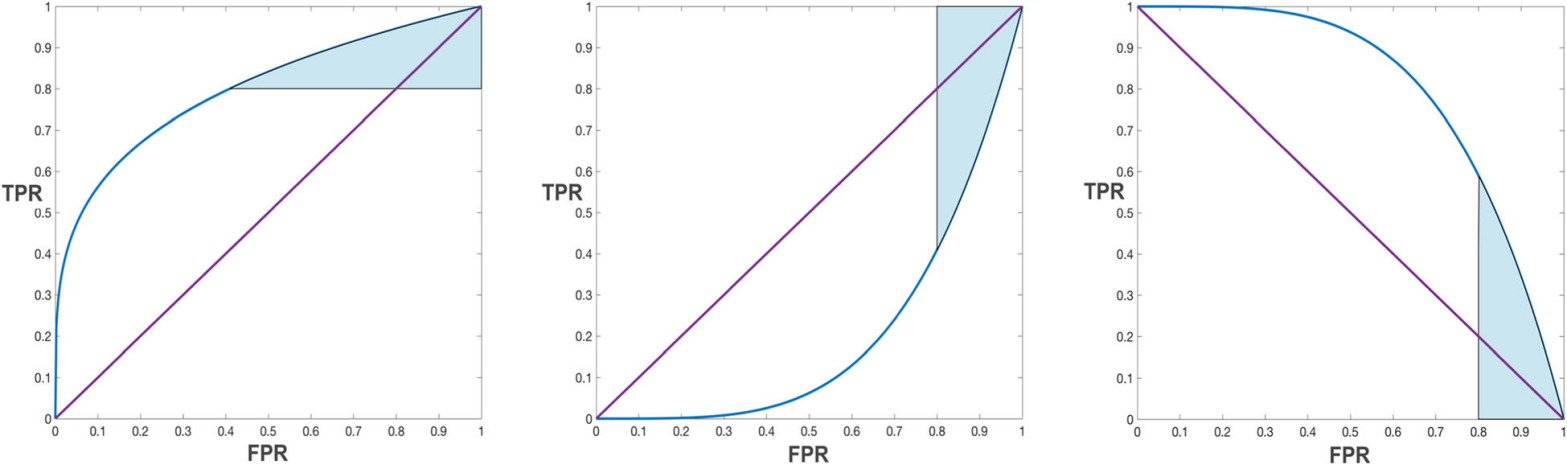
To integrate horizontally perform two simple transformations (swap the axes, flip the new vertical) and then integrate normally (vertically). The two transformations have the same effect as a 90 degree clockwise rotation

The *pAUC* is also insufficient for high prevalence data [2, 17, 18] where the top (often top-right) portion of an ROC curve is of interest (e.g., Fig. 3a). McClish [17] suggests that one could use the *pAUC* while “reversing the role of disease and non-disease”. Walter [2], suggests that the area to the right of a curve could be observed (integrated) like the original *pAUC* but would lack symmetry.

Finally, McClish provides a standardized version of *pAUC* [17].

### Review of the area under the precision recall curve (AUPRC)

The precision recall curve (PRC) and corresponding area under the PRC (AUPRC) purposefully focus on positives, with the *y* axis indicating how often a positive classifier/test result is actually positive (precision), and the *x* axis indicating how many of the actual positives the classifier/test will detect (recall). AUPRC is also called average precision (AP).

In low prevalence data, negatives are predominant in numbers and the AUPRC allows positives to be sufficiently weighted or considered despite the greater proportion of negatives. This may be useful in information retrieval, e.g., to find similar cases of a rare disease [19], however for many medical problems such as screening or diagnostic testing, negatives and negative predictive value (NPV) must be sufficiently considered at the same time since both types of errors have costs. To that end, the AUPRC may be computed a second way, separately, to focus on the negatives while largely ignoring positives. However the shortcoming of the AUPRC is that it is not comparable to the more popular ROC plot and *AUC*, it has no connection to the *c* statistic and it is reported as a two-part measure, for each class separately.

### Related work

Related work on several alternatives to the partial *AUC* are found in the literature [18, 20–22] however none of them, including the partial *AUC*, have the same three mathematical relationships (formulas) that the *AUC* has. The *AUC* is equal to concordance, average *TPR* and average *TNR*—where each aspect facilitates understanding and explanation. To the best of our knowledge, we derive the first partial measure which maintains all three relationships of the *AUC*—the “concordant partial area under the curve” (see the section by that name).

Jiang et al. [18] define a partial area index (PAI) for a range of TPR above a threshold. They compare a computer aided diagnostic (CAD) versus radiologists in the identification of benign and malignant cancers using mammograms. The authors select a sensitivity threshold of *TPR >* = 0*.*9, based on the assumption that identifying malignant cancer is more important than causing unnecessary biopsies for benign conditions. The authors find that the computer’s ROC curve is significantly higher (*p* = 0.03) than the radiologists’ ROC curve with their partial area index, whereas with the *AUC*, the difference was not significant (*p* = 0.21).

Wu et al. [22] propose a learned partial area index that learns the clinically relevant range from the subjective ratings of physicians performing a task. For the task of identifying and segmenting tumors in radiological images, the authors perform an experiment with 29 images comparing an automated probabilistic segmentation algorithm with radiologists ratings. The results highlight that in radiologic diagnosis of cancer, *FPR* is more important than *TPR*. The authors conclude that ranges of *FPR* and *TPR* can be defined based on clinical indication and use.

Related work on a partial concordance (*c*) statistic in the literature [23–26] do not correspond to partial areas in an ROC. To the best of our knowledge, we derive the first partial *c* statistic for partial curves in ROC data. Using a similar term, may cause some initial confusion among readers, but our context is sufficiently different and it is appropriate to reuse the term partial *c* statistic as it corresponds to the term partial *AUC* in our context.

**Fig. 4:**
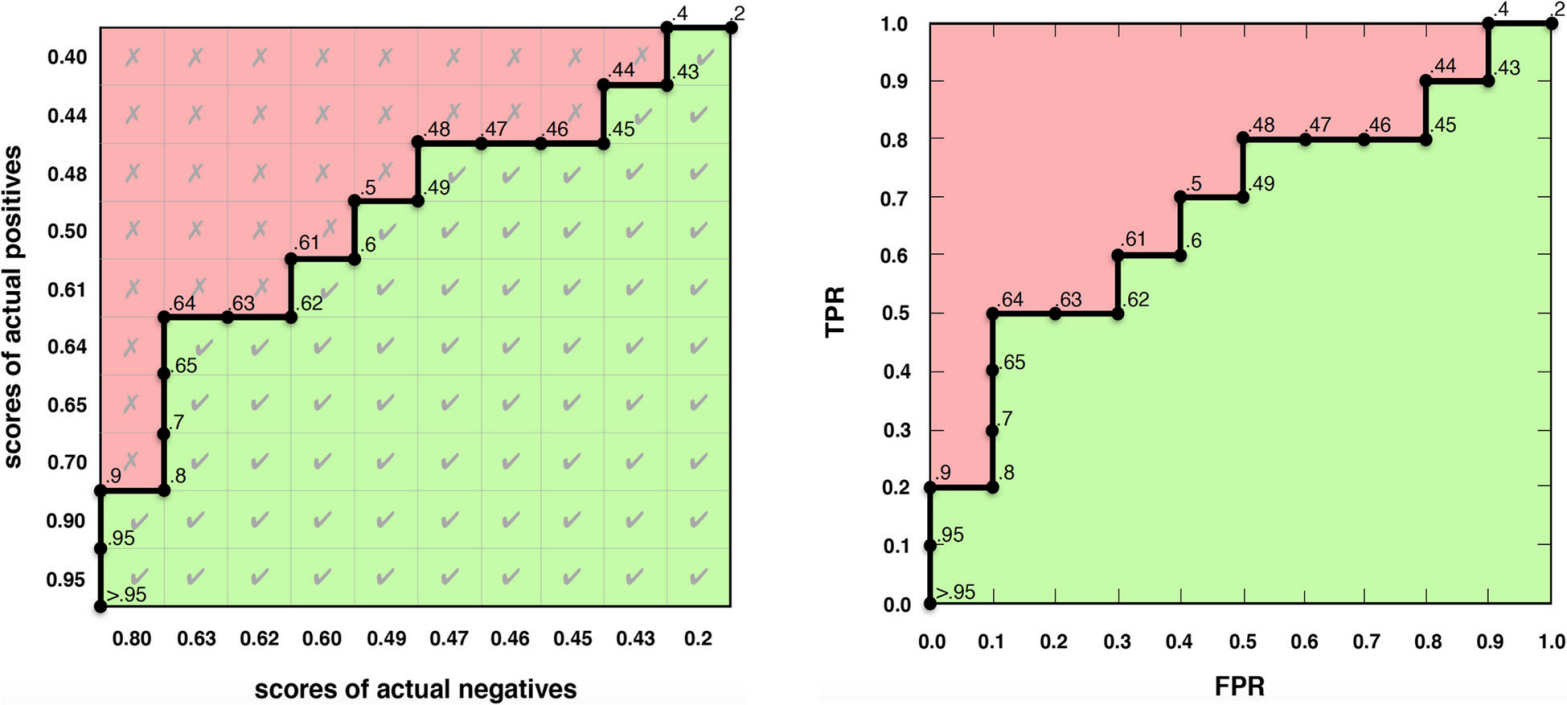
The concordance matrix and ROC plot. **a** The proposed concordance matrix visualizes how the c statistic is computed—as the proportion of correctly ranked pairs (green) out of all pairs. **b** The empirical ROC plot (above) equals the border in the concordance matrix (left), visualizing the known equivalence between the *c* statistic and the *AUC*

We develop the idea for a concordance matrix and find that Hilden [27] depicted the same idea. Placements or placement values [28, 29] are a related concept, sometimes in table/matrix form [30] but they are not ordered in the same way and they lack a key insight: geometric equivalence between empirical ROC curves and concordance as we later show (Fig. 4). Placements have been used to explain the (vertical) partial *AUC* [28], but not a combined horizontal and vertical perspective for partial measures, as in our proposed partial *c* statistic and proposed concordant partial *AUC*.

The only work with some similarity to the combined perspective of our proposed measures comes from jackknife pseudovalues [30, 31]—but its numeric perspective is not as readily translated into the ROC interpretations we seek.

## Problem statement and solution

If the *AUC*, *pAUC* and AUPRC are not fully adequate to summarize, understand and explain the performance of a classifier or diagnostic test with low prevalence data, then what do we require to rectify the situation?

We require a partial area measure that can focus on a region of the ROC that is of interest, and which has three relationships like the *AUC*—a relation to the *c* statistic, to average *TPR* and to average *TNR.*^1^

We solve the problem statement by proposing the concordant partial *AUC*, *pAUC*_*c*_ (Fig. 1b)(Table 1), as half the sum of the partial (vertical) area under the ROC curve *pAUC* [12, 13] and the horizontal partial area under the ROC curve. This sum is derived from how concordance and partial concordance are computed. All of these measures are defined in subsequent sections except for *pAUC* [12, 13] previously discussed.

### The horizontal partial area under the curve

To capture the horizontal perspective on a partial curve we define the horizontal partial *AUC* (*pAUC*_*x*_) as Walter [2] suggests, the area to the right of the curve (Fig. 3a). We refer to this as “under the curve” henceforth for consistency with the term *AUC*. We do not reuse the partial area index [18] because we must be able to select both ends of the range.

Horizontal integration uses the right border *x* = 1 as the baseline (Fig. 3a) and the distance to the ROC curve left of that to measure the true negative rate (TNR). Normally integration is defined with the *x* axis (*y* = 0) as the baseline, but if we swap the *x* and *y* axes we get *x* = 0 as a baseline (Fig. 3b). If we then transform *x* (FPR) to 1 *− x* (TNR), i.e., reverse it, or flip it about its center (Fig. 3c), we get TNR as needed and the *x* = 0 baseline becomes *x* = 1 as needed. The integration bounds remain the same (Fig. 3). We therefore define *pAUC*_*x*_ as follows:

2 $$ {pAUC}_x\triangleq {\int}_{y_1}^{y_2}1-{r}^{-1}(y) dy $$

### Concordance: the c statistic

The *c* statistic [7–9, 32] is a measure of discrimination [9, 10] that is variously referred to as the *C* statistic [10], concordance [8], the *C*-index [32, 33] and Kendall’s coefficient of concordance [25]. The concept and its equivalence to the *AUC* first arose in classification in the two-alternative force choice (2AFC) experiment [34]. It was later defined for regression and survival analysis by Harrell Jr. et al. [32]. It should **not** be confused with Hosmer and Lemeshow’s [35] *C*^ˆ^ statistic which is a measure of calibration [9].

For every possible pair of one actual positive *p*_*j*_ and one actual negative *n*_*k*_ in a test or validation set, the *c* statistic for a classifier or diagnostic test is the proportion of times when the classification score *g* (*·*) for the actual positive is greater than the score for the actual negative, i.e., is ranked correctly [36]. The formula,

3 $$ c\triangleq \frac{1}{PN}\sum \limits_{j=1}^P\sum \limits_{k=1}^NH\left(g\left({p}_j\right)-g\left({n}_k\right)\right) $$

measures the *c* statistic for data with *P* and *N* actual positives and negatives, respectively, and uses the Heaviside function *H* (*·*) to count correct ranking as 1, ties as 0.5 and incorrect ranking as 0.

It is important to note that the *c* statistic equals the area under the ROC curve (*AUC*) for ROC data with a binary outcome–but not censored data [36, 37] (e.g., survival or time-to-failure) data. In the next section, we visualize this statistic.

### The concordance matrix for ROC

We formalize the concept of a concordance matrix which depicts the *c* statistic for ROC data as a rectangular matrix of actual positives on the *y* axis versus actual negatives on the *x* axis (Fig. 4a) ordered such that scores monotonically increase toward the origin.

Hilden [27] first illustrated this concept as a probabilistic interpretation of the ROC area, using scores with the opposite meaning and order from common con- vention as in [4].

With the definition of concordance in mind from the previous section, the concor- dance matrix shows the correctly ranked pairs in concordance toward the bottom- right, incorrectly ranked pairs toward the top-left and a border in between which exactly corresponds to the empirical ROC curve (Fig. 4).

This illustrates the well-known equivalence between the *c* statistic and *AUC* [7–9, 11, 38] even though they are computed differently.

### The local c statistic (towards the partial c statistic for ROC data)

**Fig. 5:**
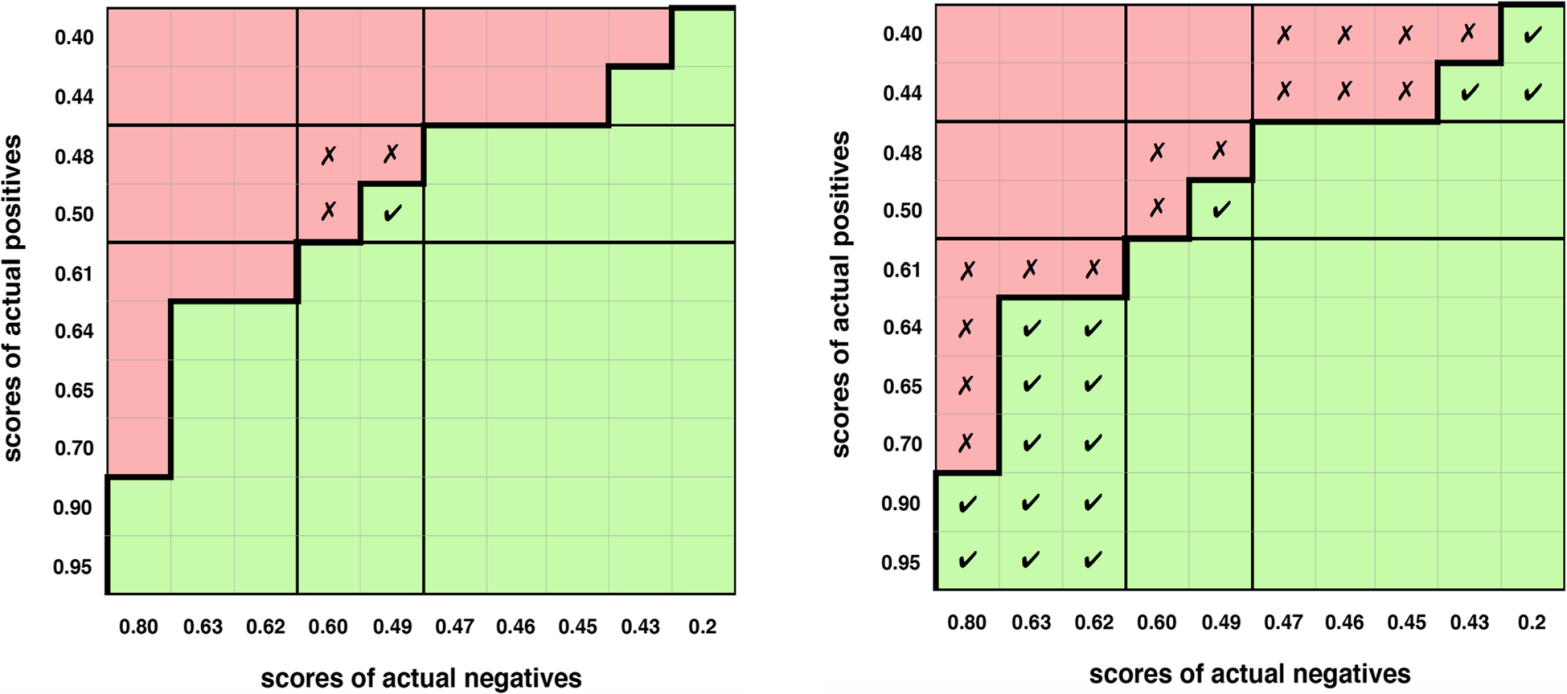
Local concordance for one versus all parts of the border. **a** Local concordance for the middle part of the concordance matrix border split into three disjoint parts. **b** Local concordance for all three parts of the concordance matrix border does not use all of the cells in the matrix

For a partial curve we first hypothesize and define a local *c* statistic (*c*_*L*_), which like the whole *c* statistic, represents the percentage of correctly ranked pairs of one actual positive with one actual negative, but is limited to the ROC data points which fall in the range of the partial curve (Fig. 5a).

This may seem to have the same meaning as the whole *c* statistic at first glance, but there is no way to relate a sum, product or weighted average of *c*_*L*_ values to the *c* statistic because it lacks information from cells in the matrix over multiple parts which comprise the whole ROC curve (Fig. 5b). The *c*_*L*_ is an incomplete view of a partial area related to the curve and its contribution to the *AUC*. Also, since the concordance matrix demonstrates an exact correspondence between *c* and *AUC*, we expect that a proper partial *c* statistic in the concordance matrix will correspond to the concordant partial *AUC* we proposed in the introduction.

### The partial c statistic for ROC data

**Fig. 6:**
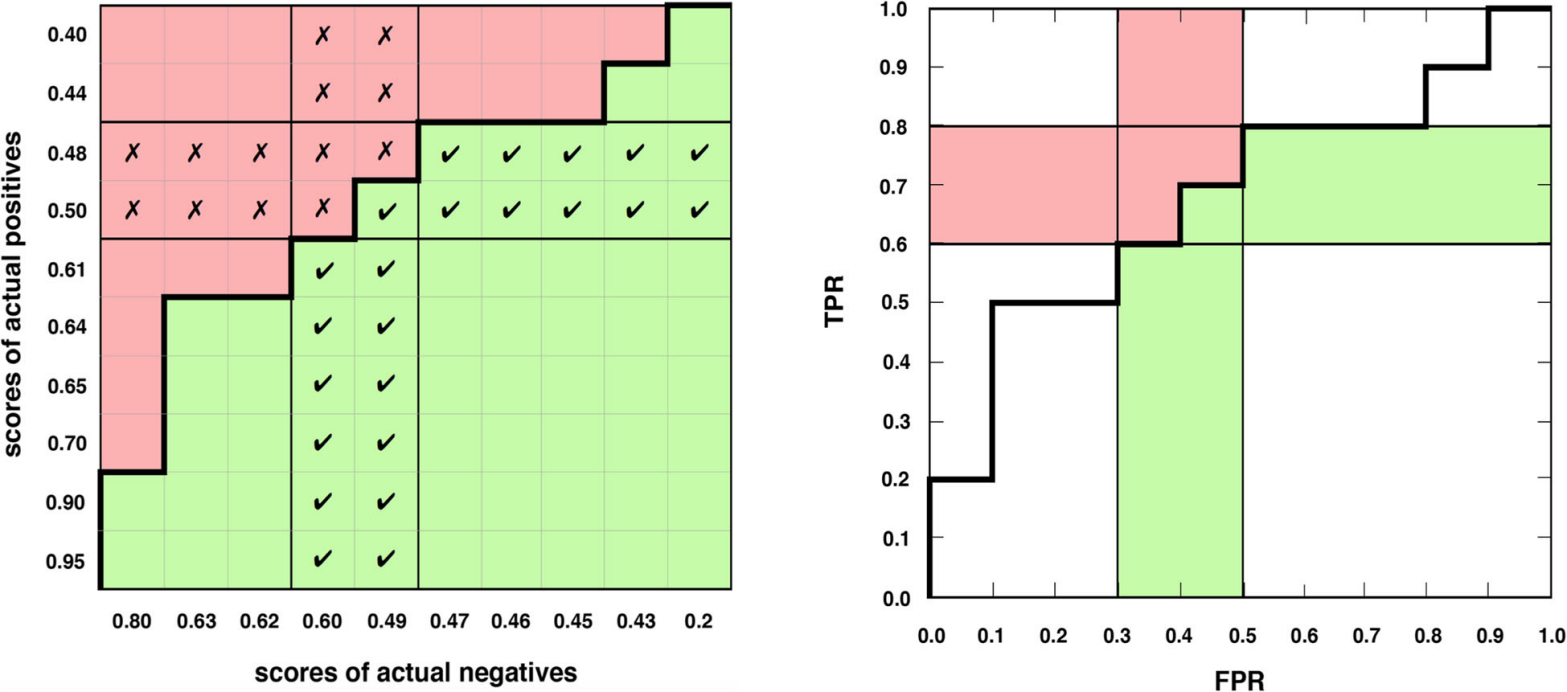
Partial concordance versus concordant partial *AUC*. **a** The partial *c* statistic for part of the concordance matrix border (or ROC curve). **b** The concordant partial *AUC* in green corresponds to the green (positive) cells highlighted in the matrix at left

There are two obvious possible ways to define a partial *c* statistic, and in the previous section we found that the first way, the local *c* statistic, is insufficient. Hence, we define the partial *c* statistic (*c*_*∆*_) in the second obvious way, to include off-diagonal interactions—and we confirm that this provides complete and accurate information. We define *c*_*∆*_ based on a set of columns and a set of rows (Fig. 6a).

In computations for both *c*_*∆*_ and *pAUC*_*c*_, there is a region of overlap that is counted twice, and division by two in the equation for *c*_*∆*_ accounts for that.

We define simplified *c*_*∆*_ for a partial ROC curve with *J* out of *P* actual positives $\left\{{p}_{1\dots J}^{\prime}\right\}$ and a subset of *K* out of *N* actual negatives $\left\{{n}_{1\dots K}^{\prime}\right\}$, *c*_*∆*_ as below. *H*(·) is the Heaviside function and *g* (*·*) are classification scores.

4 $$ \mathrm{simple}\ {c}_{\Delta }\triangleq \frac{1}{2 JN}\sum \limits_{j=1}^J\sum \limits_{k=1}^NH\left(g\left({p}_j^{\prime}\right)-g\left({n}_k\right)\right)+\frac{1}{2 PK}\sum \limits_{j=1}^P\sum \limits_{k=1}^KH\left(g\left({p}_j\right)-g\left({n}_k^{\prime}\right)\right) $$

The formula above (4) has two parts which are summed: the proportion of correctly ranked cells within a vertical and horizontal stripe (Fig. 6a). The measure may be normalized for explanation:

5 $$ {\overset{\sim }{c}}_{\Delta }\triangleq \frac{2 PN\cdotp {c}_{\Delta  }}{J\cdotp N+K\cdotp P} $$

And the partial *c* statistic over all *q* disjoint partial curves that comprise the whole curve, sums to the *c* statistic:

6 $$ c=\sum \limits_{i=1}^q{\left({c}_{\Delta }\right)}_i $$

**Fig. 7:**
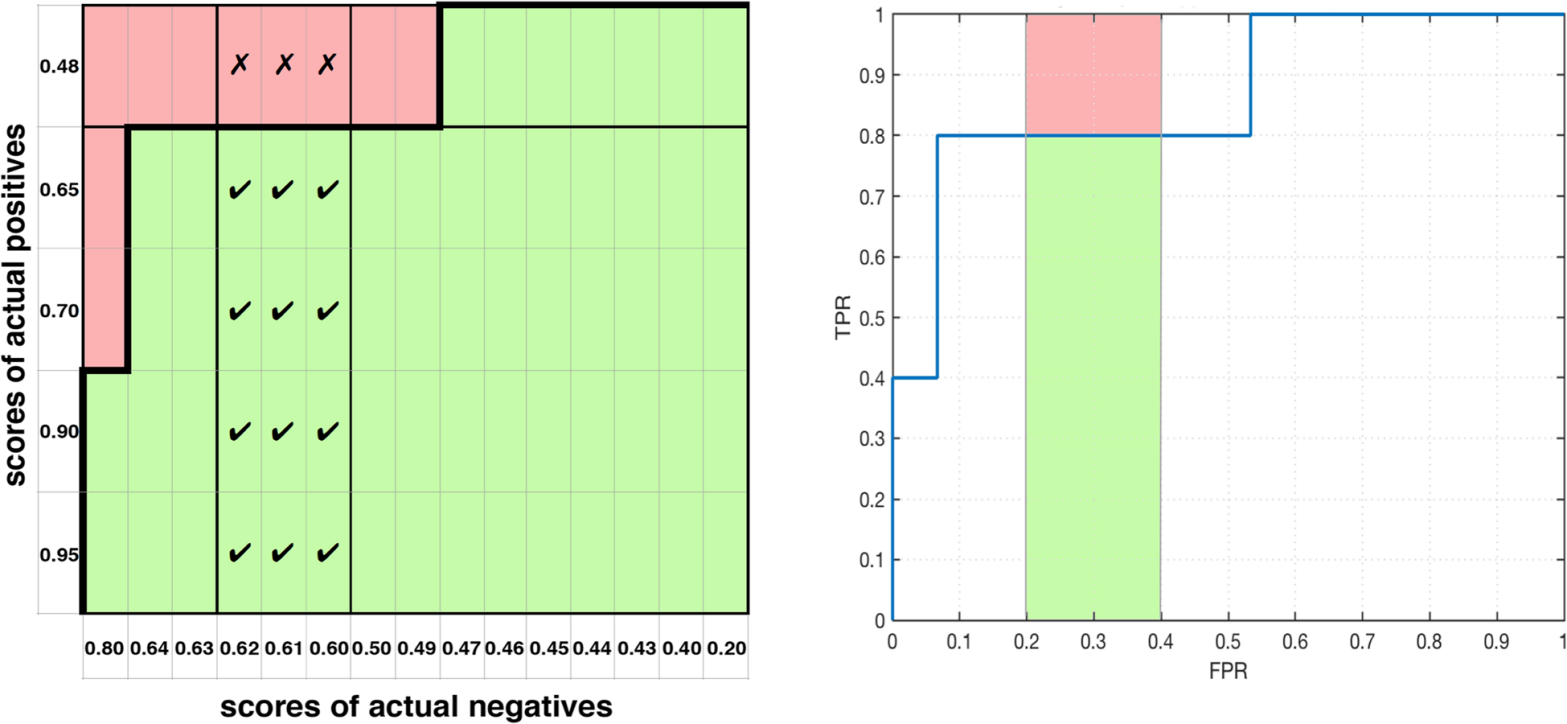
Imbalanced data and partial ROC curves without a horizontal area component. **a** Our measures work with imbalanced data—e.g, five positives to fifteen negatives. **b** Our measures also work with partial ROC curves that have no horizontal area component (or no vertical area component)

We first use the partial *c* statistic on a classic example ROC from Fawcett [4] with an equal number of positives and negatives. However, it works equally well if we use ROC data with one positive for every three negatives (as an arbitrary example) and if one (or some) of the partial curves has only a horizontal or vertical component (Fig. 7).

**Fig. 8:**
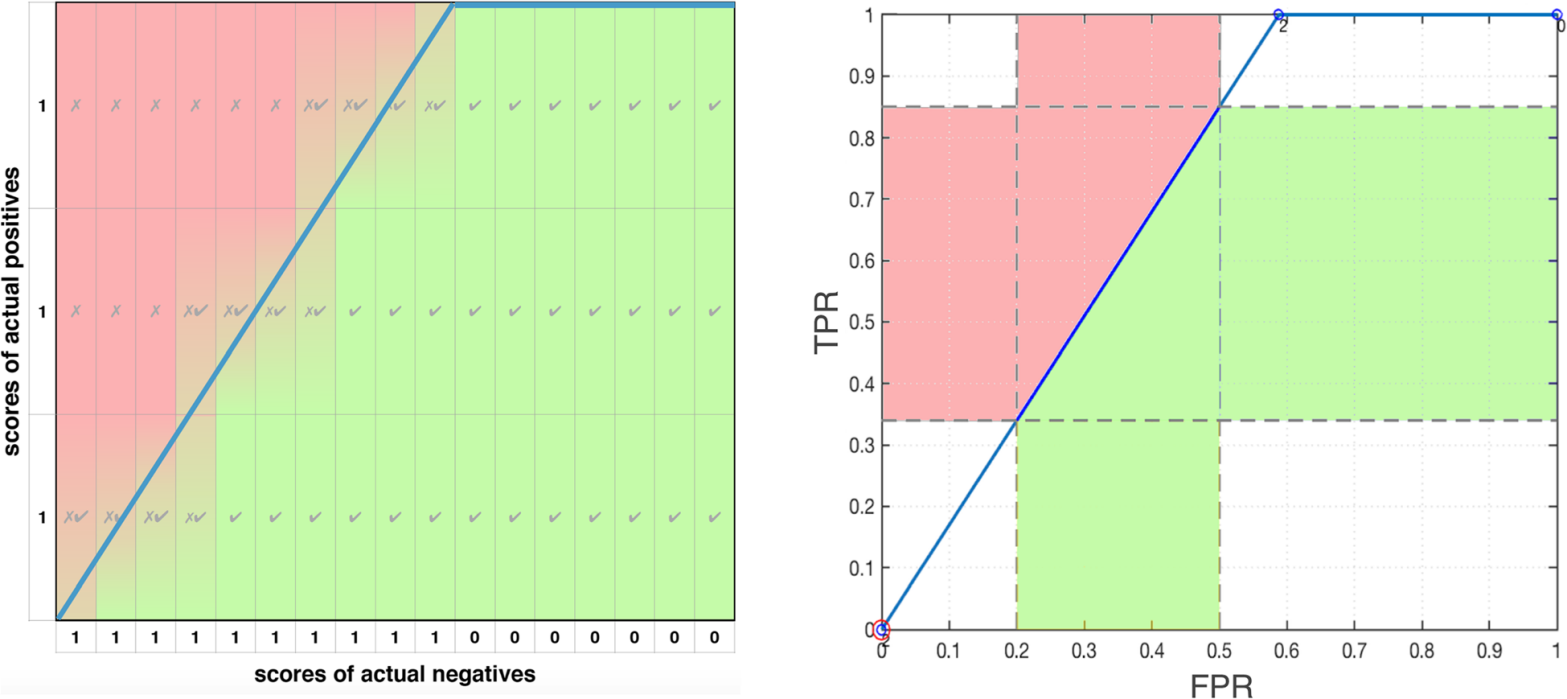
Interpolation and ties in the concordance matrix. At left, ties in score exist along both axes. At right, the partial curve’s right boundary has a height of 0.85, hence interpolation is required to compute the partial *c* statistic

The general case which the partial *c* statistic must account for, requires interpolation (Fig. 8). That is, when the partial curve has endpoints that do not match the scores of data points, we must interpolate to use a portion of a data point in calculations of the proportion for concordance. This is done by altering (4) to use partial weights for endpoints in positives in weight vector ${\underset{\_}{w}}^{+}=\left[{w}_q\right],\forall q$ and negatives in weight vector ${\underset{\_}{w}}^{-}=\left[{w}_r\right],\forall r$:

7 $$ {\displaystyle \begin{array}{c}{c}_{\varDelta}\triangleq \frac{1}{2N{\sum}_{q=1}^J{w}_q^{+}}\sum \limits_{j=1}^J\sum \limits_{k=1}^N{w}_j^{+}\cdot H\left(g\left({p}_j^{\prime}\right)-g\left({n}_k\right)\right)\\ {}+\frac{1}{2P{\sum}_{r=1}^K{w}_r^{-}}{\sum}_{j=1}^P{\sum}_k^K{w}_k^{-}\cdot H\left(g\left({p}_j\right)-g\left({n}_k^{\prime}\right)\right)\end{array}} $$

### The concordant partial area under the curve

We define the concordant partial *AUC*, *pAUC*_*c*_ (Fig. 6b) as half the sum of the (vertical) partial area under the ROC curve *pAUC* and the horizontal partial area under the ROC curve *pAUC*_*x*_ defined by *FPR* = [*×* 1*, ×* 2] and *TPR* = [*y*1*, y*2].

8 $$ {pAUC}_c\triangleq \frac{1}{2} pAUC+\frac{1}{2}{pAUC}_x $$

9 $$ =\frac{1}{2}{\int}_{x_1}^{x_2}r(x) dx+\frac{1}{2}{\int}_{y_1}^{y_2}1-{r}^{-1}(y) dy $$

This sum is derived from how concordance (3) and partial concordance (4)(7) are computed. That is, this formula is **not** arbitrarily chosen to be a sum or average—it follows how the *c* statistic and partial *c* statistic are computed as a sum with equal weighting. No other weighting will maintain equivalence with the partial *c* statistic.

Division by two is necessary in the formula to ensure that the partial *AUC* sums to *AUC* instead of 2*·AUC*. This reflects the fact that every point under the curve is integrated (or included) exactly twice. Notably, *AUC* could be computed as half the horizontal integral and vertical integral, but the *AUC* is a special case where those two integrals and areas are necessarily equal, and where average TPR and average TNR are necessarily equal [11]. Due to this redundancy, the *AUC* as a special case is computed using only the vertical integral, but our concordant partial *AUC*, is a generalization of the *AUC* to any case, partial or whole and reveals its implicit nature which contains both perspectives.

Since our concordant partial *AUC* is derived from the *c* statistic, it fulfills all expectations for summation and equality.

If we take the sum of *pAUC*_*c*_ measures for any set of partial curves which span the whole ROC curve and which are not overlapping, they sum to the *AUC* and *c* statistic. That is, if we apply a subscript, *i*, to a complete set of *i* = 1. *.*. *q* non-overlapping partial curves, the concordant partial *AUC* for each partial curve, denoted (*pAUC*_*c*_)_*i*_, has a relationship to *AUC* and *c* as follows:

10 $$ AUC=c=\sum \limits_{i=1}^q{\left({pAUC}_c\right)}_i $$

For the *i*^*th*^ partial curve, (*pAUC*_*c*_)_*i*_ is equal to (*c*_*∆*_)_*i*_:

11 $$ {\left({pAUC}_c\right)}_i={\left({c}_{\Delta }\right)}_i $$

Both measures in (11) can be normalized by dividing by the areas and proportion of cells, respectively. Also, in (9) *pAUC*_*c*_ reduces to equality with *AUC* when the partial curve is defined as the whole curve.

The concordant partial *AUC* has all three key interpretations of the *AUC*. First, it includes the *pAUC* (average sensitivity or TPR) in a way that makes its effect clear and separable from other effects (8). Second, it includes *pAUC*_*x*_ (average specificity or TNR) in a way that makes its effect clear and separable from other effects (8).

Third, it is equal to the partial *c* statistic *c*_*∆*_ (11) which is derived from concordance and the concordance matrix.

One complexity with the dual perspective of the concordant partial *AUC* is that a range along one axis, either the *x* axis (FPR) or the *y* axis (TPR), does **not** uniquely specify a partial curve for a classifier. For example, for the vertical part of a staircase ROC plot (Fig. 4b), at least two points match a value in FPR. Also, two different classifiers that share a common range specified in FPR will generally have different ranges in TPR.

Hence, if a user wishes to only specify values in FPR (similar to the *pAUC*) for convenience, then one must impose consistent choices or rules to resolve ambiguity among multiple matching points, such as the following:

1. For the first and leftmost partial curve, if there is ambiguity about:
  The left endpoint, choose the most southwest ROC point.
  The right endpoint, choose the most northeast ROC point.
2. For all other partial curves, if there is ambiguity about:
  The left endpoint, choose the most northeast ROC point.
  The right endpoint, choose the most northeast ROC point.

These rules make measurements consistent and can prevent overlap between partial curves, if desired.

## Experimental method, data and results

Our experimental method has two steps: first, we validate expected equalities among measures on four data sets; then, we validate the behaviour of measures as inequal- ities. We explain this in detail below.

In the first step we use four data sets for validation:

1. Fawcett’s classic example ROC data [4]
2. Fawcett’s example ROC data [4] modified for class imbalance
3. The Ljubljana breast cancer data set [39], and
4. The Wisconsin breast cancer data set with only 2 features [40]

The Ljubljana breast cancer data seeks to detect recurrence versus non-recurrence at 1 year after treatment.

We show the results with the Ljubljana breast cancer data set in Table 2. In all three partial curves *i* = {1…3}, the concordant partial *AUC*, *pAUC*_*c*_, and the partial c statistic, *c*_*∆*_, are equal to each other as expected, and the sums of each partial measure equal the whole measure, as expected. These equalities were validated in all four data sets.

**Table 2:** Area measures and *c* statistics are shown for 3 parts of an ROC curve *i* = {1 . . . 3} as well as the whole curve, for a classifier, a support vector machine, applied to Ljubljana breast cancer remission data. Best values per column are shown in bold font

*i*	*FPR*	*TPR*	*pAUC*	*pAUCc*	*pAUCx*	*c*∆
1	[0*.*00*,* 0*.*33]	[0*.*00*,* 0*.*84]	21*.*3%	**49***.***5**%	**77***.***7**%	**49***.***5**%
2	[0*.*33*,* 0*.*66]	[0*.*84*,* 0*.*95]	29*.*5%	17*.*4%	5*.*3%	17*.*4%
3	[0*.*66*,* 1*.*00]	[0*.*95*,* 1*.*00]	**34***.***0**%	17*.*9%	1*.*8%	17*.*9%
sum	–	–	84*.*8%	84*.*8%	84*.*8%	84*.*8%
whole	*AUC* = *c* = 84*.*8%*AUPRC*+*,−* = 72*.*2*,* 53*.*7%					

In the second step, we examine the behaviour of partial and whole measures and their meaning.

Our interpretation begins by considering the area under the curve (*AUC*) as a summary measure of a classifier’s overall performance [41, 42]. The higher the *AUC*, the closer the classifier is to being perfect in classifying actual positives and negatives *at one threshold* at or toward the top left corner. This should also be true of a normalized partial measure if it is meaningful—the higher the number, the better the classifier is overall with actual positives and negatives. However, this is not true for the normalized partial *AUC* ($\overset{\sim }{pAUC}$) when comparing different partial curves for the same classifier (Table 3) because it monotonically increases with FPR.

**Table 3:** Normalized area measures $\overset{\sim }{pAUC}$, $\overset{\sim }{pAUC_c}$ and *sPA* are shown for 3 parts of an ROC curve using a support vector machine classifier on Ljubljana breast cancer remission data. Best values per column are shown in bold font

*i*	*FPR*	*TPR*	$\overset{\sim }{pAUC}$	$\overset{\sim }{pAUC_c}$	*sPA*
1	[0*.*00*,* 0*.*33]	[0*.*00*,* 0*.*53]	64*.*6%	84*.*5%	78*.*8%
2	[0*.*33*,* 0*.*66]	[0*.*53*,* 0*.*88]	89*.*3%	79*.*8%	89*.*4%
3	[0*.*66*,* 1*.*00]	[0*.*88*,* 1*.*00]	**99***.***9**%	**90***.***1**%	**99***.***7**%

Hence McClish [17] proposes the standardized Partial Area (*sPA*). *sPA* subtracts any area under the major diagonal (considered non-informative) and then standardizes the result to the range [0*.*5*,* 1]. This removes monotonic behaviour, but the subtraction which is related to Kappa and AUK, diverges from the meaning of *AUC* and concordance. When *sPA* is computed on portions of an improper ROC curve [11, 43, 44] it can yield a negative value, which does not occur with our concordant partial *AUC* (*pAUC*_*c*_).

*pAUC*_*c*_ is a balanced measure but the leftmost partial area is the region of interest for classifying fewer positives than negatives. In some cases (Table 4), *pAUC*_*c*_ ranks classifiers like average precision (AP or AUPRC) in the leftmost area and differently from *pAUC*. AP (or AUPRC) is thought to be a good measure for imbalanced data, preferred over *AUC* [45, 46], and it is more popular measure than *pAUC*.

**Table 4:** We report the performance of four classifiers in one experiment with best values per row shown in bold font

Measures	LDA	LogR	SVM	NN	NN-SVM
Whole Area					
*AUC*	82*.*9%	77*.*1%	84.8%	**86***.***0**%	1*.*2%
*AUPRC*+	60*.*9%	53*.*5%	**72***.***2**%	71.0%	−1.2%
*AUPRC−*	54.5%	**56***.***7**%	53*.*7%	53*.*3%	−0.4%
Partial Area *i* = 1					
*sPA*	75*.*0%	69*.*2%	78.8%	**79***.***2**%	0*.*4%
*pAUC*	19*.*2%	16*.*0%	21.3%	**21***.***6**%	0*.*3%
*pAUC*_*c*_	47*.*5%	37*.*2%	**49***.***5**%	48.0%	*−*1*.*5%
Partial Area *i* = 2					
*sPA*	90.0%	82*.*2%	89*.*4%	**92***.***2**%	2*.*8%
*pAUC*	29.7%	27*.*1%	29*.*5%	**30***.***4**%	0*.*9%
*pAUC*_*c*_	18*.*5%	**22***.***9**%	17*.*4%	21.0%	3*.*6%
Partial Area *i* = 3					
*sPA*	**100**%	**100**%	99.7%	**100**%	0*.*3%
*pAUC*	**34***.***0**%	**34***.***0**%	**34***.***0**%	**34***.***0**%	0%
*pAUC*_*c*_	17.0%	17.0%	**17***.***9**%	17.0%	*−*0*.*9%
*sPA*: sum of NN-SVM					3*.*5%
*pAUC*: sum of NN-SVM					1*.*2%
*pAUC*_*c*_: sum of NN-SVM					1*.*2%

**Fig. 9:**
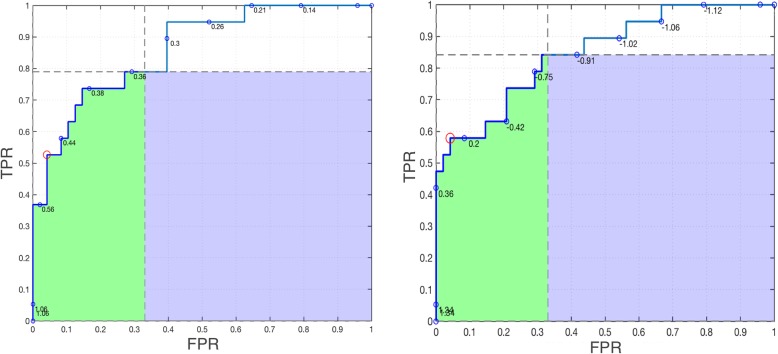
A comparison of the leftmost partial curve and area between two classifiers applied to Ljubljana breast cancer remission data. **a** Neural network (NN) ROC plot. **b** Support vector machine (SVM) ROC plot

Next we compare the performance of two classifiers. Table 4 shows that differences between neural network (NN) and support vector machine (SVM) classifiers (NN-SVM) in partial areas sum to the difference between the *AUC*. Next, consider the first or leftmost partial curve/area—this is the region of interest when there are few positives relative to negatives. Fig. 9 compares the NN and SVM classifiers. We hope that the ROC curve goes up quickly and/or stays to the left hand side, but in Fig. 9 it is difficult to tell which curve is better. The SVM curve goes up faster initially while staying left, and it ends at a higher value of TPR, resulting in more of the blue area, Also, the optimal ROC point (red circle) is better (closer to the top right) for SVM than NN. Consistent with these facts *pAUC*_*c*_ is higher for SVM. However, the NN curve goes up more quickly in the middle (*FPR≈*1*.*5) and has more green area. Consistent with the vertical perspective (the green area only, not blue) *pAUC* is higher for NN.

## Discussion

The *AUC* and *c* statistic are important standard measures and our proposed con- cordant partial *AUC* and partial *c* statistic are the partial equivalents thereto. Alternative partial measures such as *pAUC*, *sPA* and others discussed in related work (e.g., *PAI*) are not as complete nor comprehensive in their relationships to the *AUC*, *TPR* (Sensitivity), *TNR* (Specificity) and the *c* statistic.

Class imbalance in data traditionally prompted the use of the alternatives to the *AUC* including partial measures or *AUPRC*, but *pAUC*, *sPA* and *AUPRC* are biased toward positives and are each one half of a pair. *AUPRC* is paired with *AUPRC−* and *pAUC* (and *sPA* by extension) is paired with *pAUC*_*x*_. The goal is not to identify the best measure for all tasks, but to understand the meaning, limitations and proper application of each measure.

## Conclusions

We proposed a concordant partial area under the curve *pAUC*_*c*_ for ROC plots which is a foundational partial measure, and unlike alternatives, has all three of the interpretations offered by the *AUC*: a relationship to the average true positive rate, the average true negative rate (or false positive rate) and the *c* statistic (or concordance).

We also proposed a partial *c* statistic and concordance matrix which shed light on the meaning of partial areas. Finally, we showed by experiment that the behaviour of our proposed measures correctly match theory and are meaningfully interpreted.

An important contribution of this paper is to help the reader understand and explain the justification, assumptions, benefits and risks of area measures and c statistics in ROC plots. We described the risks of measures focused primarily on positives, and we proposed partial measures with desirable interpretations like their whole counterparts.

## Future work

Future work may include: demonstrating the value of the concordant partial area for balanced data and high prevalence data; comparison of our proposed measures with other measures not based on areas; and combining our proposed measures with other ROC measures and techniques.

## Data Availability

The Wisconsin [40] and Ljubljana [39] breast cancer datasets used in this paper are available in the University of California and Irvine Machine Learning Repository [47]: https://archive.ics.uci.edu/ml/datasets/Breast+Cancer+Wisconsin+(Diagnostic) https://archive.ics.uci.edu/ml/datasets/breast+cancer}

## Abbreviations

*AP*: Average precision
*AUC*: Area under the ROC curve
*AUPRC*: Area under the precision recall curve
*c*: The *c* statistic or concordance
*c*_∆_: Partial *c* statistic or partial concordance
*c*_*L*_: Local *c* statistic or local concordance
FNR: False negative rate
FPR: False positive rate, or 1-specificity, or 1-TNR
*PAI*: Partial area index
*pAUC*: Partial area under the ROC curve (i.e., vertical)
*pAUC*_*c*_: Concordant partial area under the ROC curve
*pAUC*_*x*_: Horizontal partial area under the curve (i.e., to the right)
PRC: Precision recall curve
ROC: Receiver operating characteristic
sPA: Standardized partial area
TNR: True negative rate, or specificity, or selectivity
TPR: True positive rate, or sensitivity, or recall, or 1-FNR

## Footnotes

1 Since *TNR* = 1 *− FPR,* measures in terms of average *TNR* are easily translated to measures in average *FPR* and vice-versa.
